# Models for T-large granular lymphocytic leukemia: how to mimic the cellular interplays in malignant autoimmunity

**DOI:** 10.1038/s41375-025-02553-2

**Published:** 2025-03-07

**Authors:** Hanna Klepzig, Marco Herling, Natali Pflug, Till Braun

**Affiliations:** 1 Department I of Internal Medicine, Center for Integrated Oncology (CIO) Aachen Bonn Cologne Düsseldorf, Translational Research for Infectious Diseases and Oncology (TRIO), University Hospital Cologne, Cologne, Germany; 2 Department for Hematology, Cellular Therapy, Hemostaseology, and Infectious Diseases, University Hospital of Leipzig and Cancer Center Central Germany (CCCG), Leipzig-Jena, Germany; 3 Mildred Scheel School of Oncology Aachen Bonn Cologne Düsseldorf (MSSO ABCD), Cologne, Faculty of Medicine and University Hospital of Cologne, Cologne, Germany

**Keywords:** Cancer models, T-cell lymphoma

## Abstract

T-large granular lymphocytic leukemia (T-LGLL) is a chronic lymphoproliferative disorder characterized by clonal expansions of cytotoxic T-cells. It presents with cytopenias that are not explained by the typically low leukemic burden. Notably, T-LGLL is frequently accompanied by autoimmune disorders, particularly rheumatoid arthritis (RA). As clonal T-cell expansions are also increasingly identified in autoimmune-driven conditions, better models of T-LGLL’s pathogenesis as a spectrum of (auto)antigen-driven oligoclonal hierarchies towards overt leukemic escape with associated immune dysregulations would provide details to a valuable prototype for determinants of T-cell fitness and transformation as well as T-cell instructed dysfunctions of other immune cells. Such insights would advance our concepts of cancer biology and immunology. Common molecular links between T-LGLL and autoimmune diseases include activation of JAK/STAT signaling, proinflammatory cytokine environments, and antigen-driven immune responses. Current murine models address these mechanisms rather individually: JAK/STAT based systems replicate pathway activation, cytokine-driven models simulate inflammatory conditions, and RA models often mimic antigen stimulation. However, none of these fully captures the duality of clonal T-cell expansion and the complex immune dysregulations, inherent to T-LGLL. This review examines criteria for autochthonous in-vivo T-LGLL models and evaluates existing systems, identifying their strengths, limitations, and specific representations of clinico-pathologic aspects of LGLL. Prominent transgenic models, for example, not only manipulate the T-cell compartment but also indiscriminately alter the tumor microenvironment, impeding research on the specific role of elements of the LGLL micromilieu. We propose strategies to overcome such insufficiencies of present models. Overall, our critical appraisal emphasizes the need for novel comprehensive models that more faithfully integrate the key features of T-LGLL or for models that, by featuring specific pathogenetic aspects of the disease, would supplement existing incomplete systems. We expect such new model systems to aid in better understanding the cancer-immunity interface and in assessing novel therapeutic approaches for T-LGLL.

## Introduction

T-large granular lymphocytic leukemia (T-LGLL) is a chronic lymphoproliferative disorder characterized by the clonal expansion of mature T cells [1]. Although classified as a rare disease, T-LGLL is among the most prevalent mature T-cell leukemias [2]. The oligo- to monoclonal expanded leukemic cells are post-thymic T cells, presenting in most patients a terminally differentiated effector memory CD3^+^CD8^+^ phenotype, with diminished CD5 expression and clonal restriction to the T-cell receptor (TCR) beta constant 1 (TRBC1) or TRBC2 chain [3]. A minority of patients develop CD4^+^ leukemic cells, presenting a more indolent disease course and, therefore, handled as a distinct subtype [3]. Additionally, T-LGLL is closely related to NK-LGLL, likely linked by similar pathogenic mechanisms, though the exact factors influencing the dominance of each clonal population remain unclear [4].

Clinically, patients frequently present with severe neutropenia, leading to recurrent infections, along with transfusion-dependent anemia [3]. Bone-marrow infiltration is commonly seen in T-LGLL patients, but interestingly, the severity of cytopenias does not correlate with the degree of T-LGLL infiltration, suggesting that immunogenic mechanisms, beyond those of anti-neutrophil antibodies or Coombs-positive hemolytic anemia, play a role [3]. Furthermore, T-LGLL is frequently associated with autoimmune conditions like rheumatoid arthritis (RA) or severe vasculitis [3]. A ’watch and wait’ approach is appropriate for asymptomatic patients, but our clinical experiences show that treatment has to be initiated in ~70% of patients with options remaining limited. Standard therapy includes immunosuppressive agents like low-dose methotrexate, cyclophosphamide, or cyclosporine, often combined with temporary glucocorticoids and supportive measures (e.g. transfusions). However, those treatments are often insufficient in controlling disease and symptoms; and come with relevant side effects, emphasizing, that novel strategies are highly warranted [3].

To develop new, innovative therapeutic strategies, preclinical models are of indisputable importance. T-LGLL presents at a unique intersection between cancer and immune dysregulation, exhibited by the clonal expansion of malignant T cells and the association with autoimmune mechanisms that drive clinical presentation. Current mouse models, however, fail to capture this duality of T-LGLL. As our understanding of the disease’s complex pathomechanism grows, emerging insights are reshaping our view of its biology. These evolving perspectives highlight the need for refined preclinical models that reflect this complexity. In this review, we present an updated concept of T-LGLLs pathogenesis, outline the core criteria required for effective in-vivo modeling, and critically examine existing models. We highlight the future challenges in more accurately representing the intricate interplay of immune dysregulation and malignant transformation in T-LGLL.

## Current pathogenic concept of T-LGLL

**Fig. 1:**
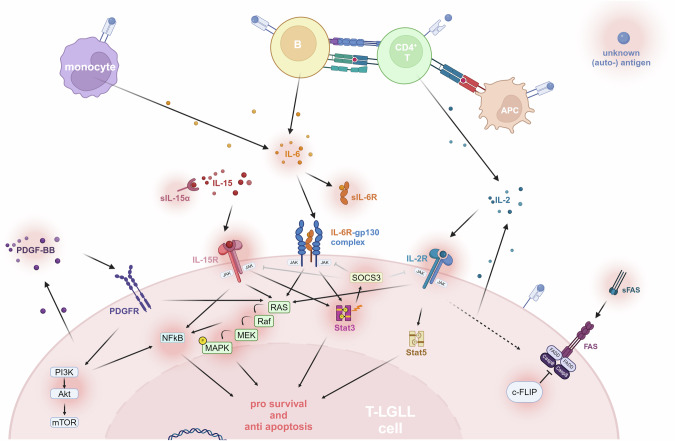
Proposed concept of T-LGLL pathogenesis. This schematic overview illustrates the pathobiological landscape of T-LGLL, highlighting key genomic, transcriptomic, and microenvironmental factors that drive T-LGLL leukemogenesis. The proposed initial event for T-LGLL is a chronic antigenic stimulation, resulting in polyclonal LGL expansion. The expansion of LGLs is further supported by inflammatory cytokines, like IL-2, IL-6, and IL-15, potentially secreted by the non-leukemic compartment, like B-cells, CD4^+^ T cells, monocytes, and other antigen-presenting cells (APC). Autocrine platelet-derived growth factor (PDGF)-BB signaling, via PI3K/Akt/mTOR, adds an additional proliferative stimulus. Clonal selection of T-LGLL expansions can be further favored by the occurrence of specific mutations, predominantly gain-of-function (GOF) mutations of *STAT3* in CD8^+^ T-LGLL and *STAT5B* in CD4^+^ T-LGLL. Constitutive activation of STAT3 is additionally achieved by diminished expression of the negative regulator SOCS3. Pathways conferring resistance to apoptosis are also shown, with increased c-FLIP levels contributing to Fas/FasL resistance. Red shadows indicate proven alteration within T-LGLL cells; dashed arrows show suspected but unconfirmed interactions, while gray arrows denote downregulated effects in T-LGLL. The figure was created with Biorender.

To critically evaluate existing mouse models, a comprehensive understanding of the pathobiology of T-LGLL is essential (see Fig. 1 for the current conceptual framework of the disease). While the central pathways and cellular interactions presented in this review focus on aspects relevant to mouse models, a more in-depth review of current pathogenetic concepts can be found in [5]. The initiating ‘event’ for the clonal expansion of LGL cells is proposed to be a chronic and persistent antigen stimulation, although no common antigen has been identified so far [3, 6]. Potentially triggered by this chronic antigen stimulation, constitutive STAT3 activation emerges as the molecular hallmark of the disease, providing survival advantages to the escaping T-LGLL clone and leading to its (pre)leukemic expansion. This activation is in 30–75% of T-LGLL patients evoked by somatic gain of function (GOF) mutations in *STAT3*, located in its Src-homology (SH) 2 domain resulting in stabilized dimerization and, thereby, enhanced activation [7, 8]. The most prevalent mutations, *Y640F* and *D661Y*, account for 60% of all mutations [7]. STAT3 activation is involved in various biological processes, including the promotion of antiapoptotic and prosurvival mechanisms, with altered transcription of target genes like *Mc1*, *c-Myc*, *cyclins D1* and *D2*, *Bcl-xl*, and *TP53* [9, 10]. Besides recurrent GOF mutations, the lack of the negative feedback mechanism by downregulation of SOCS3 further sustains constitutive STAT3 activation in T-LGLL cells [11]. *STAT5B* mutations are significantly less frequent in T-LGLL; they mainly occur in CD4^+^ αβ T-LGLL (7–66%), are less frequent in γδ T-LGLL (0–19%), and are only rarely seen in CD8^+^αβ T-LGLL or NK-LGLL [12]. Furthermore, T-LGLL outgrowth is supported by resistance to Fas/FasL mediated apoptosis, leading to escape from activation-induced apoptosis. Physiologically, Fas-mediated regulation limits the survival of antigen-stimulated T cells to maintain homeostasis. FasL binding activates the death-inducing signaling complex (DISC), which is essential for downstream signaling [13]. In T-LGLL, increased c-FLIP levels inhibit activation of the DISC and enhance resistance to Fas-mediated apoptosis despite the availability of all required components of the pathways, namely surface expression of Fas receptor and constant expression of FasL [13, 14].

Aberrant cytokine signaling is another hallmark in the pathogenetic concept of T-LGLL. Most centrally, the proinflammatory IL-15 signaling pathway, which supports survival of CD8^+^ memory T cells, is altered in T-LGLL patients. Besides elevated *IL-15* mRNA expression levels in T-LGLL cells, increased levels of sIL-15Rα were detected in T-LGLL patients-derived plasma [15, 16]. The relevance of IL-15 signaling is highlighted by promising efficacy of a first trial using a selective IL-15 inhibitor in T-LGLL [17]. Known for its dual role in T-cell survival, IL-2 stands out as another mechanism of altered T-cell signaling. Physiologically, it supports T-cell proliferation and survival in the initial phase of an immune response but later increases the sensitivity to Fas-mediated apoptosis to prevent excessive immune response. In T-LGLL, ex-vivo treatment of apoptosis-resistant T-LGLL cells with IL-2 enhances DISC formation and increases the activity of caspase-8, re-sensitizing the cells to Fas-mediated apoptosis. Additionally, upregulation of the IL-2 receptor on T-LGLL cells, and its downstream signaling activating JAK/STAT, MAPK, and NF-κB signaling, may contribute to the clonal sustenance [13, 18]. Furthermore, constitutive JAK/STAT signaling, e.g. mediated by activation of STAT3, is additionally sustained by IL-6, with both IL-6 and IL-6 receptor subunit α (IL-6Rα) reported to be elevated in T-LGLL patients [11]. Additionally, increased PDGF-BB, produced by T-LGLL cells, alongside with expression of PDGF receptors on T-LGLL cells leads to an autocrine proliferation stimulus via PI3K and Akt/Erk pathways [19].

Recently, the pivotal role of the non-leukemic immune cell compartment in T-LGLL pathogenesis was highlighted. Utilizing single-cell RNA-sequencing of T-LGLL patient-derived peripheral blood mononuclear cells (PBMCs), alterations not only in the leukemic but also the non-leukemic compartment were shown, possibly connected by various cytokines that show elevated serum levels in T-LGLL patients [6, 20]. Exemplarily, intraindividual comparisons between the antigen-specificity of T-LGLL clones and the non-leukemic T-cell compartment revealed shared target antigens, suggesting antigen-driven clonal hierarchies and expansions [6]. Monocytes, key producers of cytokines and professional antigen-presenting cells, are promising candidates for interactions in this context. In T-LGLL, the composition of monocytes shifts towards a higher proportion of non-classical and intermediate monocytes [21]. Most predicted T-LGLL cell-cell interactions are with monocytes, and many elevated cytokines can in fact be monocyte-derived [6]. Additionally, patients with T-LGLL exhibit a greater proportion of mature, terminally differentiated CD4^+^CD57^+^ T cells [6] and an imbalanced T_H_17/T_reg_ ratio[21]. The latter is a known pathomechanism in RA, highlighting this alteration as a possible link to autoimmune phenomena in T-LGLL. Finally, the success of therapeutic approaches that target non-T cells, e.g. rituximab targeting CD20 as a classic B-cell marker, further substantiates the critical role of the microenvironment in sustaining the T-LGLL clone and manifestation of clinical symptoms [22].

## Cell line models of T-LGLL

To advance our understanding of T-LGLL pathogenesis and facilitate therapeutic discoveries, ex-vivo models are critical, as they bridge the gap between molecular profiling and in-vivo complexity, enabling controlled manipulation and analysis. The MOTN-1 cell line is the most commonly used T-LGLL-like system. It exhibits a CD3^-^CD4^+^CD8^-^ TCR-incompetent immunophenotype, which is exclusively observed in CD4^+^ T-LGLL and does not accurately represent typical T-LGLL cells. Additionally, its dependence on IL-2 further complicates its applicability, as the IL-2 supplementation alters activation state and cytokine profiles within its mono-culture, thereby further restricting its utility [23].

## Key criteria of an accurate T-LGLL-like mouse model

Efforts to develop new T-LGLL-like mouse models must go beyond replicating isolated aspects of the disease; instead, they should focus on creating a comprehensive model that ideally accurately reflects the full complexity of T-LGLL’s pathology. To resemble the unique position of T-LGLL as the prototypic model for a ‘malignant (auto)immune-synapse’, an ideal pre-clinical model should capture (i) an oligo- to monoclonal expansion of (ii) terminally differentiated effector memory CD8^+^ T-cells accompanied by (iii) signs of inflammation, with both phenomena present in an (iv) indolent model. (v) Alterations should resemble pathogenetic hallmarks of T-LGLL, e.g. STAT3 GOF mutations. Additionally, (vi) the broader immune cell compartment should be genetically (in the experimental sense) unadulterated, to facilitate studies on a tumor microenvironment (TME) that is predominantly shaped by the T-cell clones or that itself shapes the outgrowing T cells in a genetically unbiased trajectory. Ideally, (vii) the lead findings of T-LGLL in humans, such as cytopenias, should be reflected.

**Fig. 2:**
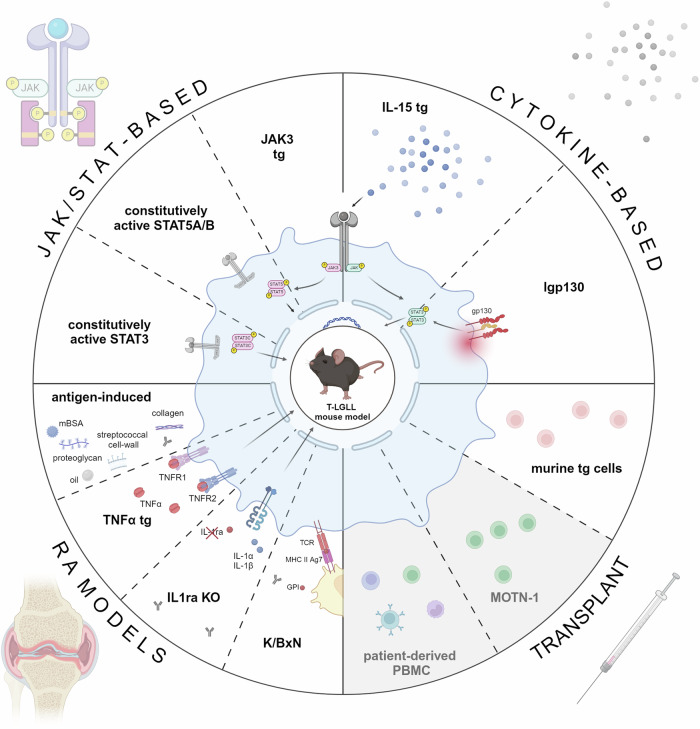
Overview of murine models resembling T-LGLL or associated autoimmune diseases. Murine T-LGLL-like models are either based on aberrant JAK/STAT signaling, focusing on constitutive activation of JAK3, STAT5A/B, and STAT3, (upper left corner), or proinflammatory cytokine signaling, involving enhanced IL-15 expression or constitutive activation of the IL-6 family receptor glycoprotein 130 (gp130, upper right corner). In addition, murine models of the commonly associated rheumatoid arthritis (RA) are displayed (lower left corner), divided into models generated through antigen injection (left side) and those developed via transgenic modifications (right side). Besides transgenic mouse models, transplantable models of T-LGLL-like disease are conceivable (lower right corner), involving syngraft transplantation of transgenic, leukemic T-cell populations, as well as xenografts of the T-LGLL-like cell line MOTN1 or patient-derived peripheral blood mononuclear cells (PBMCs). Models with gray backgrounds represent proposed strategies, but have not yet been established. The figure was created with Biorender.

Several murine models that resemble T-LGLL, at least parts of its symptom complex, or models of (auto)immune conditions that are central part of T-LGLL’s pathophysiology, have been established. A detailed overview of all models reviewed can be found in Tables 1,  2, details are provided in Supplementary Table 1, and an overview is presented in Fig. 2. Our outlines are centered on capturing the most common T-LGLL phenotype, which is characterized by the expansion of CD8^+^ terminally differentiated memory T cells.

**Table 1:** Murine models resembling mature T-cell leukemia/lymphoma based on cytokine and constitutive JAK/STAT activation.

		IL-15 tg	Gp130 (lgp130^CD4/wt^)	STAT5B tg (*hSTAT5B*^*N642H*^)	Hyperactive STAT5A tg (*STAT5A*^*S710F*^)	JAK3 tg (*JAK3 M511I*)	STAT3 tg (*STAT3*^*T716M*^*)*	STAT3 tg (*STAT3*^*K658N*^)
**MODEL**	**Murine disease**	Aggressive, leukemic CD8^+^ TEM/NK-cell expansion in setting of chronic inflammation	T_H_17 driven multiorgan autoimmunity and inflammation	Expansion of (a) CD8^+^CD25^+^ mature aggressive T-cell leukemia (b) immature DN1 (when crossed to RAG2 KO mice)	Aggressive CD8^+^ mature, cytotoxic T-cell leukemia	Aggressive immature CD3^-^/TCRβ^-^ TdT^+^ T-cell leukemia	Aggressive, polyclonal NKG2D^hi^ effector CD8^+^ T-cell expansion	Aggressive, polyclonal NKG2D^hi^ effector CD8^+^ T-cell expansion
**MODIFICATIONS**	**(Transgenic) modifications**	IL-15 overexpression by elimination of posttranscriptional checkpoints	Constitutive signaling via forced gp130 dimerization and activation	Constitutive expression of human *STAT5B*^*N642H*^ (GOF mutation)	Hyperactive STAT5A variant (cS5A^hi^)	Constitutive active JAK3 M511I	*STAT3*^*T716M*^ GOF mutation	*STAT3*^*K658N*^ GOF mutation
	**Mutation type/treatment**	Germline (MHC class I D^d^ promoter)	Targeted (loxP flanked, ROSA26 locus)	Germline (VAV1 promotor)	Germline (VAV promotor)	Transplant JAK3 M511I expressing hematopoietic stem cells	Germline	Germline
	**Spont./ transp./ind**.	Spont.	Spont.	Spont.	Spont.	Transp.	Spont.	Spont.
**PHENOTYPE**	**Strain**	FvB	C57BL/6	C57BL/6	C57BL/6	BALB/c	C57BL/6	C57BL/6
	**Weight loss**	Y	Y	NA	NA	NA	Y	Y
	**Spleno-megaly**	Y	Y	Y	Y	Y	Y	Y
	**Lymphadenopathy**	Y	Y	NA	Y	NA	Y	Y
	**WBC count**	↑	↓	↑	↑	↑	↑	↑
	**Median overall survival**	130-196 d	45d-69d	40-100 d	175-315 d	100-200 d	hom.: >350 d	het.: >315 d hom.: >175 d
**LEUKEMIA**	**Leukemic expansion**	Y	N	Y	Y	Y	Y	Y
	**Clonality relevant sub-population**	Y	N	NA	NA	Oligoclonal	Polyclonal	Polyclonal
**IMMUNITY**	**Immuno-competent**	Y	Y	Y	Y	Y	Y	Y
	**Auto-antibodies**	NA	NA	NA	NA	NA	NA	NA
	**Infiltration of lymphocytes in peripheral organs (other than joints)**	Y	Y	Y	NA	NA	Y	Y
	**Inflammation**	Skin	Liver Heart Spleen	NA	NA	NA	Skin Joints	Skin Joints
**CYTOKINES**	**Cytokines, increased (↑) or otherwise relevant in pathogenesis**	IL-15 ↑	IL-6 ↑ sIL-6Rα ↑ IL-17a ↑ TNFα ↑ IFNγ ↑ IL-10 CCL5/RANTES	NA	NA	NA	NA	NA
**IMMUNOPHENOTYPE**	**All T**	↑	↑	↑	NA	NA	NA	NA
	**Immature T**	NA	NA	↑	NA	↑	NA	NA
	**CD8**^**+**^ **T**	↑	↑	↑	↑	↑	↑	↑
	**CD8**^**+**^ **memory T**	↑	↑	↑	↑	NA	↑	↑
	**CD4**^**+**^ **T**	NA	↓	NA	NA	NA	NA	NA
	**Other immune cell populations**	B cells↓	B cells ↑ (LN) B cells ↓ (spleen)	B cells ↑ (spleen) NK cells ↑	B cells ↑ (spleen) NK cells ↓	NA	Neutrophils ↑	Neutrophils ↑
	**Original reference**	[24]	[27, 28, 67]	[29]	[37]	[34]	[41]	[41]

**Overview presenting main features of different murine models**. ↑ indicates an increase; ↓ a decrease. When referring to cell populations, these symbols denote changes in the total number of cells unless otherwise specified as a percentage (%). If changes are restricted to specific compartments, the relevant compartment is indicated in brackets. Detailed references and additional information are provided in Supplementary Table 1.

*CD* cluster of differentiation, *d* days, *DN* double negative, *GOF* gain of function, *gp130* glycoprotein 130, *het.* heterozygous, *hom.* homozygous, *ind.* induced, *JAK* Janus kinase, *KO* knock-out, *LN* lymph nodes, *MHC* Major Histocompatibility Complex, *N* no, *NA* not available, *NK* cell Natural Killer cell, *ROSA26* Reverse Oriented Splice Acceptor, Clone 26, *STAT* Signal transducer and activator of transcription; spont. spontaneous, *TCR T-cell receptor* TEM terminal effector memory, *tg* transgenic, *TNFα* tumor necrosis factor, *transp.* transplant, *wt* wildtype, *Y* yes.

**Table 2:** Murine models resembling rheumatoid arthritis.

		Proteoglycan-induced arthritis (PGIA)	Streptococcal cell-wall arthritis	Collagen-induced arthritis (CIA)	Antigen- induced arthritis (AIA)	Oil-induced arthritis (OIA)	TNF-α tg	IL-1ra KO	K/BxN tg
**MODEL**	**Murine disease**	Immune-mediated joint disease	Biphasic, immune-mediated joint-disease *Acute phase*: T-cell independent *Chronic phase:* T-cell dependent	Chronic T-cell driven immune-mediated joint disease	Acute, locally limited, self-limiting antigen-specific T-cell driven joint disease	T-cell driven, self-limiting polyarthritis	Chronic, erosive, T- and B-cell independent polyarthritis	Chronic, T-cell driven inflammatory polyarthro-pathy	Severe T_h_2-driven inflammatory arthritis
**MODIFICATIONS**	**(Transgenic) modifications**	None	None	None	None	None	Increased TNFα-transcript stabilization	Deletion exons of IL-1ra	Transgenic TCR and MHC class II allele Ag7
	**Mutation type/treatment**	i.p. cartilage proteoglycan	i.p. peptidoglycan-polysaccharide polymers	i.d. type II collagen in complete Freund’s adjuvant	e.g. s.c./i.a. methylated bovine serum albumin	i.d. injection of incomplete Freund’s adjuvant	Germline	Germline	Germline
	**Spont./ transp./ind**.	Ind.	Ind.	Ind.	Ind.	Ind.	Spont.	Spont.	Spont.
**PHENOTYPE**	**Strain**	BALB/c	Lewis rats	e.g. DBA/1	e.g. C57BL/6 BALB/c	DA rats	C57BL/6	BALB/c	C57BL/6xNOD
	**Weight loss**	NA	NA	NA	NA	NA	Y	NA	NA
	**Spleno-megaly**	NA	NA	Y	NA	NA	NA	Y	Y
	**Lymph-adenopathy**	NA	NA	Y	NA	NA	NA	Y	NA
	**WBC count**	NA	NA	NA	NA	NA	NA	NA	NA
	**Median overall survival**	NA	NA	NA	Recovery 21 d post-injection	Recovery 45 d post-injection	84-98 wk (tg197)	NA	NA
**LEUKEMIA**	**Leukemic expansion**	N	N	N	N	N	N	N	N
	**Clonality relevant T-cell population**	NA	NA	NA	NA	Y	NA	NA	NA
**IMMUNITY**	**Immuno-competent**	Y	Y	Y	Y	Y	Y	Y	Immunocompromised
	**Auto-antibodies**	Y	NA	Y	Y	N	NA	Y	Y
	**Infiltration of lymphocytes in peripheral organs (other than joints)**	NA	NA	NA	NA	N	Y	N	N
	**Inflammation**	Joint	Joint	Joint	Joint	Joint	Joint Liver Kidney Small intestine	Joint	Joint
**CYTOKINES**	**Cytokines, increased (↑) or otherwise relevant in pathogenesis**	IFN-γ IL-6 IL-1β	NA	IL-1β TNF-α ↑ IFN-γ ↑ IL-6	IL-6	IL-2 IFNγ TNFα	hTNFα	IL-17 IL-6 TNFα COX2	IL-1 TNFα
**IMMUNOPHENOTYPE**	**All T**	NA	↓	↑ (joint)	NA	NA	NA	Not altered	↓
	**Immature T**	NA	NA	NA	NA	NA	NA	NA	NA
	**CD8**^**+**^ **T**	NA	NA	↑ (joint)	NA	NA	NA	Not altered	NA
	**CD8**^**+**^ **memory T**	NA	NA	NA	NA	NA	NA	NA	NA
	**CD4**^**+**^ **T**	NA	NA	↑ (joint)	NA	Essential for pathogenesis	NA	Not altered	↑ (joint)
	**Other immune cell populations**	NA	B cells ↓ Activated B-cell compartment % Monocytes↑	B cells (PB, LN) ↑ Activated B-cell compartment % Myeloid cells ↑ (PB, LN)	NA	Neutrophils ↑	B-cell independent disease	No alteration in T:B ratio ↑ IgG ↑ IgE Neutrophils↑ (joints)	B cells ↑ Hypergammaglobulinemia
	**Original reference**	[68]	[69]	[70]	[71]	[72]	[73]	[59]	[56]

**Overview presenting main features of different murine models**. ↑ indicates an increase; ↓ a decrease. When referring to cell populations, these symbols denote changes in the total number of cells unless otherwise specified as a percentage (%). If changes are restricted to specific compartments, the relevant compartment is indicated in brackets. Detailed references and additional information are provided in Supplementary Table 1.

*CD* cluster of differentiation, *d* days, *GOF* gain of function, *i.a.* intra-articular, *IL-1ra* IL-1 receptor antagonist, *ind.* induced, *i.d.* intradermal, *i.p.* intraperitoneal, *KO* knock-out, *LN* lymph nodes, *MHC* Major Histocompatibility Complex, *N* no, *NA* not available, *s.c.* subcutaneous, *spont.* spontaneous, *TCR* T-cell receptor, *tg* transgenic, *TNFα* tumor necrosis factor, *transp.* transplant, *wk* weeks, *Y* yes.

## Murine models mimicking T-LGLL-like cytokine signaling

First established in 2001, the transgenic IL-15 mouse model, overexpressing murine IL-15 by transgenic elimination of posttranscriptional checkpoints under the control of an MHC class I D^d^ promoter, is still one of the most commonly used T-LGLL-like mouse models [24]. The alteration results in the manifestation of a variety of diseases. In line with its physiological effect, experimental overexpression of murine IL-15 leads to the expansion of NK cells and CD8^+^ T cells accompanied by an overall chronic inflammation, manifested in skin symptoms such as whole body alopecia and lymphocytic infiltration in peripheral organs, e.g. peritoneum and lungs. Increased white blood cell counts can be detected as early as 3 weeks of age. In ~70% of mice, the developing disease is characterized by progressive whole body alopecia and other skin manifestations and only moderately increased white blood cell counts, resembling symptoms of cutaneous T-cell lymphoma (CTCL) [25]. In the remaining 30% of mice, a disease sharing many features with human NK- or T-LGLL develops; approximately half of these mice present NK1.1^+^CD122^+^CD3^−^ leukemic cells, therefore classified as NK-LGLL-like leukemia, while the remainder resemble CD8^+^ T-LGLL-like disease [26]. The malignant nature of these cells was confirmed by transplantation and successful engraftment in sublethally irradiated SCID mice, and clonality was detected in 50% of the CD8^+^ T-cell populations [26]. The subgroup developing a leukemic CD8^+^ clone is well established as a T-LGLL model, meeting most of the criteria defined previously (e.g. clonality, associated autoimmune-mediated symptoms). However, these mice show an aggressive course with a median animal survival of 28 weeks, in contrast to the non-aggressive nature of human LGLL. In addition, when studying the TME of this model, the non-conditional nature of the transgene needs to be considered.

An alternative approach for achieving constitutive STAT3 activation, without relying on direct gain-of-function (GOF) mutations, is the lgp130 model. This model constitutively expresses active gp130, engineered by replacing its extracellular domain with the human c-Jun leucine zipper (leucine zipper plus gp130, lgp130), which results in enforced dimerization. By placing it under the ROSA26 locus and a loxP-flanked transcriptional stop cassette, targeted lgp130 expression can be achieved across nearly all cell types [27]. Gp130 lies upstream of STAT3 and is responsible for downstream signaling of the IL-6 receptor complex, therefore resembling both the characteristic constitutive STAT3 activation and chronic cytokine stimulation in T-LGLL. Lgp130^CD4/wt^-mice, created by crossing with CD4 Cre mice, exhibit lgp130-expression in CD4^+^ and CD8^+^ T cells, due to their common double-positive precursor cell. This results in multi-organ autoimmunity [27], senescence, and premature aging [28]. Cytopenias, splenomegaly, lymphadenopathy, thymic involution, and massive end-organ damage are shaping the phenotype of diseased animals, which present a visible growth retardation starting from birth [27, 28]. Importantly, the mice present a strongly altered composition of the T-cell compartment. Although the CD8^+^ T-cell compartment shows the strongest expansion, a shift to an increase in terminal effector memory T cells, and an upregulation of activation markers, the main drivers of the autoimmune inflammation are T_H_17 cells, which are promoted in their proinflammatory effect by dysfunctional T_reg_ function [27]. Interestingly, the changes that occur in this model are in line with the alteration of the CD4^+^ T cell compartment observed in T-LGLL [21], shedding light on IL-6 as one possible main mechanism for CD4^+^ T-cell alterations. However, the lgp130 model in its current form does not sufficiently resemble a leukemic disease but rather a model of autoimmunity and inflammation, mainly reflected by non-clonal alterations of the T-cell compartment. In addition, the mice only have a median overall survival of 69 days. Although genetic *gp130* lesions are not reported in T-LGLL, they mimic in mice hyperactive IL-6 signaling and constitutive STAT3 activation, both central alterations in T-LGLL pathogenesis.

## Murine models mimicking T-LGLL-like JAK/STAT activation

Besides single-cytokine based models, JAK/STAT signaling as one common downstream effector pathway of the severely altered cytokine input in T-LGLL emerged as a potential strategy to develop T-LGLL-like murine models.

STAT5B was the target in some studies. *STAT5B* mutations are predominantly found in CD4^+^ T-LGLL, a disease subset that is typically associated with a more indolent course. In contrast, *STAT5B* mutations are rare in CD8^+^ T-LGLL but, when present, are strongly correlated with a more severe and aggressive disease phenotype [12]. Transgenic insertion of human *(h)STAT5B*^*N642H*^ under control of the *VAV1* promoter, leading to constitutive expression of hyperactive *STAT5B* in all hematopoietic cells, promotes a dominant aggressive CD8^+^CD25^+^ mature post-thymic T-cell leukemia, accompanied by coexisting less prominent expansions of immature double negative (DN) 1 (CD4^-^CD8^-^) T cells [29] and of the CD56^+^ T-cell compartment [30]. The expansion of CD8^+^ T-cells becomes detectable between 6 and 8 weeks of age, marking the onset of disease progression. This is followed by a rapid exacerbation of symptoms, culminating in end-stage disease by 8 to 9 weeks of age [29–31]. Notably, transplantation of both CD8^+^ T cells and double positive (CD4^+^CD8^+^) T cells leads to the development of CD8^+^ lymphomas, whereas the blockade of T-cell development at the DN3 stage accomplished by crosses to the recombination deficient RAG2^–/–^ background leads to a T-cell acute lymphoblastic leukemia (T-ALL) like disease, characterized by expansion of immature T cells. These findings stress the role of STAT5B in T-cell leukemic transformation, effective from early development stages [31]. Another model uses constitutively active murine STAT5B, generated by single nucleotide modifications (His-299→Arg and Ser-711→Phe) expressed in the T- and B-cell compartment under control of a *lck/Eµ* promoter/enhancer cassette. In line with the hSTAT5B^N642H^ model, changes in the T-cell compartment occur and are mainly characterized by proliferation of memory CD8^+^ T cells and T_reg_ cells. Phenotypically, mice present predominantly skin lesions [32]. Notably, a monoclonal progenitor B-cell population develops at lower incidence in this model [33]. Overall, genetic targeting of STAT5B signaling has the potential to trigger a CD8^+^ disease in mice that shows some features of T-LGLL. In detail, the hSTAT5B^N642H^ model resembles an aggressive mature T-cell malignancy (although no analysis of clonality was performed) but does not adequately address the indolent disease signature of T-LGLL, characterized by autoimmunity, inflammation, and clonal evolution. In general, manipulation of STAT5B leads to diverse outcomes and various murine disease(s), reflecting its central role in hematopoietic cell development, however, limiting its suitability as an approach to create a T-LGLL-like model.

Constitutive active JAK3, provoked by transplantation of retroviral transduced hematopoietic progenitor cells to express *JAK3 M511I*, the most common *JAK3* mutation in T-ALL, leads to development of an aggressive immature T-cell malignancy. Although in the initial phase, an increase in CD8^+^ T cells can be seen, TdT^+^ DN T cells prevail in the later stages of the disease. When transplanted, these mice present with increasingly more aggressive phenotype per transplant generation, characterized by massively enlarged spleen and thymus, accompanied by infiltration of TdT^+^ DN T cells into bone marrow, lymph nodes, spleen, and thymus [34]. Retroviral insertional activation of JAK1 in OT-1 cells leads to outgrowth of JAK/STAT activated CD8^+^ mature T-cells in RAG^-/-^ recipients without revealing a striking degree of overlap with human T-LGLL [35].

A high degree of structural similarity (90% amino acid congruence) and functional overlap between STAT5A and STAT5B implicates STAT5A as a potential target in the development of murine T-LGLL leukemia [36]. Introducing a hyperactive *STAT5A*^*S710F*^ variant under control of the *VAV1* promoter [37], as well as overexpression of wild-type (wt) *STAT5A* in the lymphoid compartment by using the *H-2K*^*b*^ promoter [38], leads to development of a CD8^+^ T-cell leukemia, with the *STAT5A*^*S710F*^ model closely resembling T-LGLL cells with an activated memory phenotype [37]. Both models show phenotypic similarities, as diseased mice present prominent enlargement of the spleen and lymph nodes [37, 38], accompanied by invasion of the leukemic T cells in peripheral organs [38]. Conversely, lymphoid-specific insertion of the GOF *STAT5A*^*S711F*^, leading to enhanced phosphorylation without elevated expression of STAT5A, triggered B-cell lymphomas with low incidence. Even though phenotypic manifestation, dominated by splenomegaly and lymphadenopathy in aged diseased mice was similar to the *STAT5A*^*S710*^ model and the *STAT5A*^*wt*^ overexpressing model, invasion of cells belonging to the B-cell lineage was detected [39]. Although of phenotypic fidelity, models based on manipulated STAT5A bear the limitation that no *STAT5A* mutations have been identified in T-LGLL patients and that, in contrast to STAT5B, STAT5A is of minor importance in human hematopoietic cell development [36]. CRISPR/Cas9-mediated germline introduction of either *K658N*, a mutation detected in one T-LGLL patient [40], or *T716M*, the most common *STAT* mutation in the STAT3 GOF syndrome [41], leads to a murine T-LGLL-like disease that displays expansion of a polyclonal NKG2D^high^ effector CD8^+^ T-cell population [41]. Successful bone marrow transplantation of these expanded cells in Rag^-/-^ mice confirmed their malignant nature. Phenotypic features are more severe in homozygous mice, reflected by a shorter overall survival. Lead symptoms are linked to inflammation, as the mice present with inflamed joints, skin, and ringtails. Although from this specific model, no data on the effect in other cell compartments are available, the germline mutation is most likely also affecting other *STAT3*-expressing cells, as *STAT3* mRNA expression is present in virtually all immune cell subpopulations [42]. Thus far, this model best resembles human T-LGLL, given the prominent polyclonal population of effector CD8^+^ T cells, signs of autoimmunity (joint inflammation, alopecia), and a not too aggressive clinical course with an overall animal survival of > 50 weeks [41]. However, given the germline origin of the *STAT3* GOF mutations and the ubiquitous expression of this protein, the TME can neither be properly evaluated nor therapeutically targeted, representing the major limitation of this model.

Interestingly, in other studies, insertion of *Y640F*, one of the most common *STAT3* mutations in T-LGLL, did not lead to the development of a T- or NK-lineage disease [43, 44]. Instead, mice that receive *STAT3*^*Y640F*^-transduced mouse-derived hematopoietic stem cells develop a non-transplantable myeloproliferative-neoplasm like disease, characterized by infiltration of mature myeloid cells in bone marrow and spleen, as well as presentation of leukocytosis [43]. These models use different highly experimental techniques, such as treatment of mice with 5-FU prior to isolation of the bone marrow [44] or in-vitro culture of the bone marrow cells over several days during ex-vivo manipulation [43]. Bone marrow transplanted mice only partially regain a fully functional immune system, and with this concerns persist that such a highly experimental setting would resemble the natural pathogenesis of T-LGLL.

Although alteration of the JAK/STAT pathway is an intriguing and promising approach to generating a T-LGLL-like murine model, the variability in outcomes from existing STAT manipulation raises questions about whether altering this pathway is an effective, predictable approach to creating a T-LGLL-like mouse model. In addition, these diverse effects underscore the intricate role of STAT signaling in lymphocyte development, revealing that dysregulated STAT signaling may serve as a shared pathomechanism driving concurrent alterations in both the B- and T-cell compartments. Key factors to consider when aiming at establishing a murine T-LGLL model, are the specifically targeted cell compartment and the effects of manipulations on both activation and protein expression levels.

## Murine models of T-LGLL-associated autoimmune disorders

T-LGLL is frequently associated with a spectrum of autoimmune disorders, of which RA is the most common[1]. Several mechanisms underlying each of these conditions may contribute to a facultative, but common pathogenetic path with T-LGLL, regardless whether the autoimmune condition preceded and promoted the outgrowth of T-LGLL or whether the leukemic clonal T-cell preexisted and initiated a symptomatic autoimmune condition. A particular constellation is obviously needed because our clinical experience shows that a significant proportion of autoimmune diseases do not show clonal T cells or T-LGLL, while a proportion of ∼30–40% of T-LGLL shows no laboratory or clinical signs of autoimmune phenomena, if taken aside the cytopenias in their yet-to-be resolved mechanisms. T-LGLL shares with RA the concept of chronic antigen stimulation, dysregulated immune cell compartments, and dependence on similar cytokine pathways, including IL-6 signaling. RA is primarily characterized by an imbalance in synovial T_H_17/T_H_1 and T_reg_ function, but CD8^+^ T cells are increasingly recognized as significant contributors to the local and systemic inflammation [45]. Notably, RA patients displaying anti-citrullinated protein antibodies, show clonal expansion of cytotoxic T cells, leading to an increased ratio of this subpopulation [46]. In addition, B-cells contribute to the disease by production of autoantibodies and different cytokines, and within their function as potent antigen presenting cells, leading to activation of other immune cells and shaping the immune response in RA [47]. Given the clinical and mechanistic overlap of RA with T-LGLL, RA-like mouse models offer promising approaches for optimizing T-LGLL-like models.

Several models mimicking RA use active immunization by injection of exogenous antigens to mimic the initiating stimulus. The collagen-induced arthritis (CIA) mouse model is one of the most common models in RA research and relies on intradermally injected type-II collagen to initiate an immune response. This model manifests as a chronic, T-cell-driven arthritic disease, accompanied by generation of autoantibodies and cartilage degradation. The inflamed joints show type-II collagen-specific CD4^+^ T cells and an increased number of CD8^+^ T cells [48–50]. B cells in the CIA model display an increased ratio and enhanced activation status [51]. The complex systemic immune reaction in CIA mice is further shaped by various proinflammatory cytokines, including TNF-α, IFN-γ, displaying significantly increased plasma levels, and IL-6, crucial for pathogenesis [52, 53]. The K/BxN mouse model provides a different method of sustaining chronic antigen stimulation, leading to development of chronic T-cell driven immune-mediated erosive joint disease, resembling human RA. Here, a transgenic T-cell receptor recognizes glucose-6-phosphoisomerase as an autoantigen, with the model relying on MHC-II (I-A^g7^) for disease development. The transgenic insertion is functional in a majority of T cells by usage of cassette vectors placing transgene-derived mRNA under control of the natural TCRα and -β promoter [54]. Diseased mice develop a polyclonal B-cell activation alongside the production of autoantibodies. The crucial role of these autoantibodies in the pathogenesis is highlighted by successful transplantation of the disease by serum transfer to non-transgenic mice. An increased CD4:CD8 T-cell ratio and a sustained effect of CD4^+^ T cells on B-cell activation suggest a supporting role of the CD4^+^ T-cell compartment in the pathogenesis of the joint disease [55, 56].

Other modeling approaches alter key cytokine pathways to create a proinflammatory signature. In the transgenic tumor necrosis factor (TNF) -α mouse model, TNFα expression is upregulated by increasing transcript stability. Different TNF-α overexpressing mouse strains exist, discriminated by the number of transgene copies and cytokine expression levels. The exact phenotype, particularly the severity and time to onset of symptoms, depends on the number of transgenes, exemplary the 3647 TNF-α line, containing one copy of the transgene, develops a milder form of arthritis whereas the 197 TNF-α line, containing five copies, displays a more aggressive phenotype [57]. These mice develop chronic erosive polyarthritis, accompanied by systemic effects, manifesting in chronic inflammation of liver, kidneys [58], and small intestine, alongside general growth retardation [53]. Interestingly, the preserved development of erosive arthritis in RAG-1^-/-^ mice crossed with the transgenic TNF-α mice suggests a T- and B-cell-independent pathogenesis [57].

The IL-1 receptor antagonist (IL-1ra) knock-out model uses an enhanced effect of IL-1 by depletion of all exons of its natural antagonist IL-1ra. This leads to manifestation of a dominant chronic, T-cell-driven inflammatory polyarthropathy with autoantibodies [59]. No alterations in the CD4:CD8 ratio and the T:B cell ratio are described, suggesting either alteration in both or none of these compartments. The crucial role of the T-cell compartment in disease onset is proven by the transfer of the disease by T-cell transplantation into immunodeficient nude mice [59, 60].

Murine models developed to resemble RA are, in their current form, unsuitable as models for T-LGLL. While these approaches create a pro-inflammatory background that induces systemic alterations in the immune compartment, no clonal T-cell expansion has been detected in any of the models examined.

## Transplantable models

Transplant models provide a way to bypass some limitations imposed by globally (cell lineage unrestricted) expressed mutations and enable the introduction of human immune cell compartments into mice. Transplants can originate from (a) cell lines, (b) patient-derived cells, such as PBMCs or specific cell populations, or (c) murine cells derived from other mouse models.

So far, no successful transplantation of MOTN-1, the most widely used T-LGLL-like cell line, has been reported. This is most likely due to the need for immune deficient or immunocompromised mice to avoid host-versus-graft reactions, raising concerns whether successful long-term survival of the IL-2 dependent cell line can be maintained in vivo.

Transplanting patient-derived cells, such as bone-marrow or PBMCs, into immunodeficient mouse strains to create patient-derived xenograft (PDX) models represents a promising strategy for studying immune cell compartment dynamics and disease progression. However, establishing a humanized mouse model for T-LGLL is particularly challenging due to the disease’s indolent nature, which complicates successful engraftment. Effective T-LGLL modeling may rely on transplanting peripheral blood cells from well-characterized T-LGLL patients. This approach, while promising, faces the added challenge of donor selection based on a prototypical T-LGLL phenotype and successful engraftment in mice, enabling an accurate representation of disease mechanisms in the host.

## Relationship of models for T-LGLL to those of NK-LGLL

While this review primarily focuses on T-LGLL, it is important to acknowledge its clinical relative, NK-LGLL, which shares several pathogenetic mechanisms despite being a distinct entity. Both diseases are categorized within the spectrum of mature T-cell and NK-cell leukemias in the current 5^th^ WHO classification, with NK-LGLL having been recoined as such from its term ‘chronic lymphoproliferative disorder of NK cells’ of the 4^th^ edition [2]. Clinically, both diseases are indolent and frequently associated with cytopenias and autoimmune phenomena, although these manifestations are often more severe in T-LGLL [61]. Pathogenetically, they are linked by common features such as chronic antigen stimulation, resistance to apoptosis, and constitutive activation of survival signals [62]. However, NK-LGLL and T-LGLL differ significantly in their mutational landscapes. *STAT3* mutations, the molecular hallmark of T-LGLL, are less frequent in NK-LGLL [61], while *TET2* variants are rather characteristic for NK-LGLL [63]. Moreover, the more frequent association of T-LGLL with rheumatoid arthritis reflects distinct mechanisms of interaction of the leukemic clone within the broader immune cell network [64]. These histogenetic and pathogenetic differences between both entities implicate that faithful models for T-LGLL should more specifically consider the unique molecular foundations of this disease.

Nonetheless, the overlapping pathogenetic mechanisms of these two diseases are reflected by the co-occurrence of NK- and T-cell proliferations in some of the previously outlined models. For example, IL-15 overexpression induces NK- and T-cell leukemias at equal proportions [24], while *STAT5B*^*N642H*^ mutations in murine hematopoietic cells lead to T- or NKT-cell expansions [65]. In humans, a small cohort study revealed clonal T-cell populations in 50% of NK-LGLL patients at diagnosis, with 15% reclassified as T-LGLL over time due to the more predominant expansion of a mature clonal T-LGLL population, further suggesting overlap and interconnection between these two entities [66].

## Strategies to overcome current limitations

Many existing models depend on genetic alterations driven by promoters leading to hematopoietic-cell-specific expression. However, manipulating various cell lineages, e.g. by the use of *VAV* promoters, fails to adequately capture the T-cell-specific mutational landscape characteristic of T-LGLL. Achieving more targeted genetic modifications in T cells is feasible using T-cell-specific promoters such as *lck*, while even greater precision in specifically targeting CD8^+^ T cells can be accomplished through the Cre/loxP system.

Another strategy to obtain T-cell restricted mutations could involve the transplantation of mouse-derived cell populations. This approach offers several advantages: it allows researchers to control disease severity by adjusting the number of transplanted cells, e.g. adapting an otherwise aggressive model like the IL-15tg mouse model, and lowering the number of animals required compared to traditional breeding schemes. Furthermore, it enables studies of isolated cell subpopulations in an otherwise unaltered cellular environment, especially valuable for cells obtained by insertion of germline mutations like in the *STAT3* GOF model.

A logical approach to integrating both leukemic expansion and the inflammatory aspects in one murine model would be to involve intercrossing models that exhibit each disease component, e.g. established RA models such as CIA combined with *STAT3* GOF mice. This strategy would replicate the duality of malignancy and autoimmunity in a single model. However, there is a significant risk that such an approach could lead to an overly artificial model. While it may successfully display features of both malignant transformation and immune dysregulation, it may still fail to accurately capture the protracted chronic disease course and the shared pathogenic mechanisms that would naturally link these two components.

One promising approach to induce the expansion of an oligo- to monoclonal T-cell population is the introduction of antigen-specific T cells. For example, the OT-1 mouse with its transgenic monoclonal ovalbumin (OVA)-directed TCR on the predominant CD8^+^ T-cell population could serve as a platform. By providing a targeted antigen stimulus (i.e. the cognate OVA peptide), one could trigger local or systemic expansion of CD8^+^ T cells (e.g. in crossed animals with site-specific OVA-transgenic alleles), that gain increasing autonomy by the second hit represented by a STAT3 mutation, e.g. when cross-bred with *STAT3* GOF mice.

## Conclusion

This review highlights the key aberrations in T-LGLL’s multifactorial pathogenesis and establishes essential requirements for murine systems to more accurately model the main aspects of this disease. We outline that current models helped to dissect single traits of T-LGLL’s biology and clinical presentation. However, given their limitations to resemble specific disease aspects, no existing model fully replicates the disease’s complexity or meets all necessary requirements for a comprehensive model including (i) clonal T-cell expansion, (ii) T-LGLL’s immunophenotype, (iii) autoimmune phenomena, (iv) indolent manifestations, (v) pathogenetic hallmark alterations of T-LGLL, (vi) a specifically instructed TME, and (vii) cytopenias. Addressing these challenges requires model-specific adjustments, as well as broader strategies to enhance model fidelity. It is also important to note that possibly several initiating scenarios (e.g. auto-antigen driven) funnel in a common track of T-LGLL, hence, can not be captured in one single T-LGLL model.

Refining T-LGLL models is critical for advancing research in the field. By developing more sophisticated models, we can enhance our understanding of the disease and its mechanisms. Ultimately, these improvements will also contribute to the identification of more effective treatment strategies for T-LGLL.

## Supplementary information

Supplementary Table 1
